# *Weinmannia marquesana* var. *angustifolia* (Cunoniaceae), a new variety from the Marquesas Islands

**DOI:** 10.3897/phytokeys.4.1605

**Published:** 2011-07-12

**Authors:** David H. Lorence, Warren L. Wagner

**Affiliations:** 1 National Tropical Botanical Garden, 3530 Papalina Road, Kalaheo, HI 96741 USA; 2 Department of Botany, MRC-166, National Museum of Natural History, Smithsonian Institution, P.O. Box 37012, Washington, DC 20013-7012

**Keywords:** Conservation, Cunoniaceae, French Polynesia, Marquesas Islands, *Weinmannia*

## Abstract

*Weinmannia marquesana* F. Br. var. *angustifolia* Lorence & W. L. Wagner, **var. nov.**, a new variety with narrow, simple leaves endemic to Tahuata, Marquesas Islands (French Polynesia) is described and its affinities and conservation status are discussed. It is similar to the other two varieties of this species by having simple leaves, but this new variety has much narrower leaf blades, and it resembles *Weinmannia tremuloides* in having narrow leaf blades but differs by having simple, not trifoliolate leaves.

## Introduction

Brown (1935) described the simple-leaved Marquesan endemic species *Weinmannia marquesana* F. Br. (Cunoniaceae) based on collections from Nuku Hiva, Ua Huka, and Ua Pou. He recognized the nominate variety as var. *typica* F. Br. and simultaneously described var. *glabrata* F. Br. to accommodate glabrous or nearly glabrous collections from Hiva Oa and Fatu Hiva. In his revision Bernardi (1964) recognized *Weinmannia marquesana* in a broad sense without infraspecies. Fosberg and Sachet (1972) subsequently subsumed *Weinmannia marquesana* under *Weinmannia parviflora* G. Forst., a species from the Society Islands, and recognized Brown's two taxa as *Weinmannia parviflora* vars. *marquesana* and *glabrata*. They also described the new variety *Weinmannia parviflora* var. *myrsinites* Fosberg & Sachet from Hiva Oa.

In their revision of *Weinmannia* in the Society, Marquesas, and Austral Islands (French Polynesia) Hopkins and Florence (1998) described a distinctive new trifoliolate species, *Weinmannia tremuloides* H. C. Hopkins & J. Florence, endemic to Fatu Hiva. They recognized two varieties of *Weinmannia marquesana* F. Br. (vars. *marquesana* and *myrsinites*) and synonymized both of Brown's varieties under *Weinmannia marquesana* var. *marquesana*. Following their circumscription, the widespread var. *marquesana* is characterized by its larger elliptic or ovate leaves with blades (2.5–)3.3–7.5 × (1–)1.3–4.4 cm and occurs on the major islands Nuku Hiva, Ua Huka, Ua Pou, Hiva Oa, and Fatu Hiva, whereas the second variety, var. *myrsinites* (Fosberg & Sachet) H. C. Hopkins & J. Florence, has smaller leaves with blades 1.6–2.7(–3.3) × 0.7–1.7 cm and is endemic to Hiva Oa.

Hopkins and Florence (1998) cited three specimens of a narrow-leaved variant occurring on Tahuata under var. *marquesana* without further discussion, although noting that *Weinmannia marquesana* is a variable species “with some intermediates between the two named varieties." Further collections of this distinctive variant from Tahuata show that it differs from typical var. *marquesana* by its narrowly elliptic to oblong elliptic leaves 2.5–5.8 × 0.4–1.2 cm which taper at both ends. The floral and fruit morphology of this variant fit within the dimensions known for *Weinmannia marquesana*. As it is the only *Weinmannia* taxon to occur on Tahuata and does not intergrade with either of the two previously recognized varieties in leaf morphology, we recognize and describe it as a new variety.

## Systematics

### Weinmannia marquesana F. Br. angustifolia

Lorence & W. L. Wagner var. nov.

urn:lsid:ipni.org:names:77112691-1

http://species-id.net/wiki/Weinmannia_marquesana_angustifolia

Figs 1 2 

#### Latin.

*Ab* Weinmannia marquesana *var.* marquesana *atque* W. marquesana *var.* myrisnites *foliis unifoliatis anguste ellipticis vel oblongo-ellipticis 2.5–5.8 × 0.4–1.2 cm differt.*

#### Type.

**Marquesas Islands:** Tahuata: Ridge between Amatea & Haaoiputeomo, summit crest of island, 914 m elevation, 12 July 1997 [fl, fruit], S. P. Perlman, K. R. Wood, and J.-P. Luce 15992 (holotype: US!; isotypes: BISH!, MO!, P!, PAP!, PTBG!).

#### Description.

*Shrub or small tree* 2–3 m tall, leafy twigs 0.8–1 mm in diam., terete, sparsely strigose with pale brown hairs when young, glabrate, reddish brown and lenticellate when fresh. *Leaves* opposite, unifoliate, blade narrowly elliptic to oblong elliptic, 2.5–5.8 × 0.4–1.2 cm, costa strigulose on both surfaces, glabrescent above; secondary veins 8–10 pairs, red or yellow when fresh, visible above, higher order venation reticulate, prominulous and visible to tertiary beneath, margins finely serrate, thickened, with 8–14 teeth on each side; petioles 1.5–8 × 0.7–0.8 mm, narrowly winged, strigulose, red when fresh; stipules rounded, 0.3–0.7 mm long, apex obtuse, strigulose, thick, persistent. *Inflorescences* 3.5–5.5 × 2–5 cm, usually trichotomous and consisting of 3(5) racemes, sessile or on slender peduncle 10–15 mm, the axes strigulose, red when fresh, each raceme with 25–40 flowers; bracteoles reduced or absent; *flowers* on pedicels 0.8–1.7 mm long, calyx lobes 0.6–0.9 × 0.5–0.6 mm, broadly ovate, obtuse, sparsely strigulose or glabrous without, deciduous in fruit; petals broadly ovate, 1–1.4 × 0.7–0.9 mm, apex obtuse; *male flowers* with stamens twice as long as pistil, filaments 2–2.5 mm, anthers ovoid, 0.4 mm, ovary 0.6–0.7 mm, style 0.2 mm, stigma bilobed and slightly thickened; *female flowers *with stamens subequal to pistil, filaments 1–1.2 mm, anthers ovoid, 0.2 mm, abortive, ovary 1 mm, strigulose, styles 2, 0.6–0.8 mm, stigma bilobed, papillose, and slightly thickened. *Infructescence* 5–7 × 5.5–6.5 cm; *capsules* narrowly obovoid, 4–5 × 1.5–2 mm (excluding the styles), strigulose, the persistent styles 1 mm. *Seeds* narrowly oblong- ellipsoid, c. 0.8–1 mm, comose with tuft of hairs 0.3–0.4 mm at each end.

#### Distribution.

Known only from the summit ridge of Tahuata, Marquesas Islands.

#### Ecology.

This new variety occurs in windswept native wet evergreen shrubland or low wet forest on ridge crests from 620 to 850 m elevation, with *Crossostylis biflora* J. R. Forst. & G. Forst.*, Freycinetia arborea* Gaudich.*, Metrosideros collina* (J. R. Forst. & G. Forst.) A. Gray*, Polyscias marchionensis* (F. Br.) Lowry & G. M. Plunkett, tree ferns including *Alsophila tahitensis* Brack., and numerous pteridophytes and bryophytes in the understory. Collected in flower in July and September and in fruit in July.

#### Conservation status:

Exact size of the population unknown, although this variety is said by collectors to be “locally common" or “abundant" (four collections made at 750 to 850 m elevation) or “somewhat rare" (one collection made at 620 m elevation). However, *Weinmannia marquesana* var. *angustifolia* is known only from a single locality on a single island with a total area of occupancy of less than ca. 5km2. The suitable habitat on Tahuata (ca. 61 km2) is indicated as an endangered environment, threatened by human activity (deforestation and fire), feral animals, and invasive plants, reducing the extent of the forest.We recommend placing this variety in the IUCN **Endangered** (EN) category B2a, B2b (i–iii): B2 total area of occupancy less than 500 km2; B2a, a single population known; B2b (i–iii), continuing habitat decline inferred.

#### Specimens examined.

**Marquesas Islands:** Tahuata: Ha'aoiputeomo: near the summit of Amatea on north facing ridge  850 m elevation, 9°56'36" S, 139°5'5" W, 3 Jul 2003, L. M. Dunn 198 (P, PAP, PTBG, US); Haaoiputeomo, summit ridge and highest peak on the island , 927 m, 12 July 1997, S. P. Perlman, K. R. Wood 15964 (BISH, MO, P, PAP, PTBG, US). Amatea, W of antenna, S facing slope over Hanatetena, 793 m, 13 July 1997, S. P. Perlman, K. R. Wood, J.-P. Luce 15982 (MO, PAP, PTBG, US). Ridge between Amatea & Haaoiputeomo, summit crest of island , 823 m, 12 July 1997, S. P. Perlman, K. R. Wood, J.-P. Luce 15983 (MO, NY, PAP, PTBG, US). Trail from Amatea to Moteve, above Haaoipu Bay, to NE of Hanatetena, top of ridge crest, W facing slope, 808 m, 17 July 1997, S. P. Perlman, K. R. Wood, J.-P. Luce 15995 (AD, MO, NY, P, PAP, PTBG, US). Summit of ridge above Vaitahu, near Haaoiputeomo, on ridge near antenna, along ridge crest between Vaitahu & Hanatetena, 835 m elevation, 1 September 1995, S. P. Perlman, K. R. Wood, J. P. Luce 14911 (BISH, MO, P, PAP, PTBG, US). Summit of ridge above Vaitahu, near Haaoiputeomo, on ridge near antenna, along ridge crest between Vaitahu & Hanatetena, 823 m, 1 September 1995, S. P. Perlman, K. R. Wood, J.-P. Luce 14918 (BISH, MO, P, PAP, PTBG, US). Tahuata: Vaitahu, crête d'Amatea, début de la montée raide vers la partie haute, 620 m, 10 April 1975, P. A. Schäfer 5499 (BISH, K, PTBG, US). De Hamatea (750 m) à la crête centrale de l'île, 750–850 m, 26 May 1975, J.-C. Thibault 60 (BISH, PAP, PTBG, US). Haaoiputeoma, near satelite dish, NE from Vaitahu to summit ridge; wind swept ridge, 610–762 m, 1–2 September 1995, K. R. Wood 4431 (BISH, MO, P, PAP, PTBG, US). Haaoiputeoma, near satelite dish, NE from Vaitahu to summit ridge; along wind swept rim, 610–762 m, 1–2 September 1995, K. R. Wood 4438 (AD, BISH, MO, P, PAP, PTBG, US).

**Figure 1.**
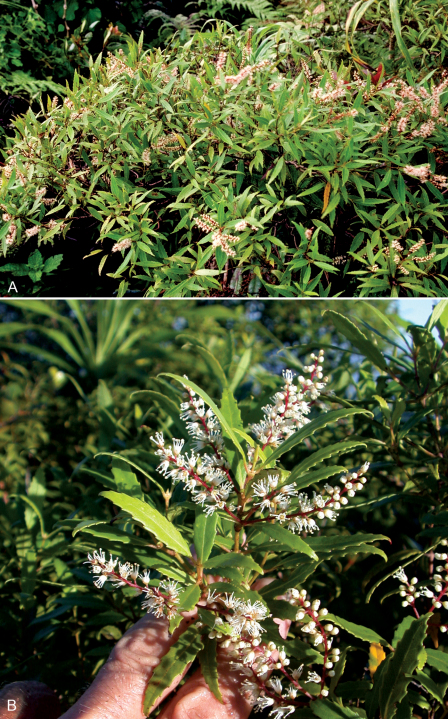
*Weinmannia marquesana* var. *angustifolia* Lorence & W. L. Wagner. **A** habit (Perlman 14911, S. Perlman photo), **B** branchlets with male flowers (Perlman 14911, K. Wood photo).

**Figure 2.**
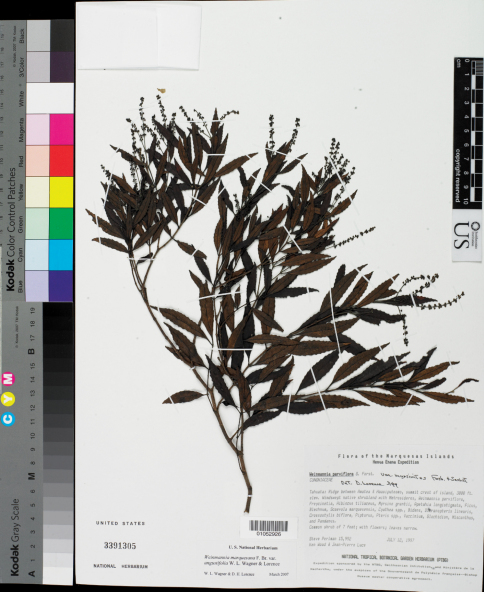
*Weinmannia marquesana* var. *angustifolia* Lorence & W. L. Wagner (Perlman et al. 15992, holotype US*).*

## Supplementary Material

XML Treatment for Weinmannia marquesana F. Br. angustifolia
